# Supplementary material for: Cloning, expression and characterization of a chitinase from Paenibacillus chitinolyticus strain UMBR 0002
Source: PeerJ. 2020 May 5;8:e8964. doi: 10.7717/peerj.8964 (PMC7207210; doi:10.7717/peerj.8964)
Supplement: Supplemental Information 2 [file peerj-08-8964-s002.pdf]

```
CLUSTAL O(1.2.4) multiple sequence alignment


WP_138185770.1      MYNTKPLFGMRKHAMRIFLLFIGLL--VLMSSSFWPGRVLGSRTVAAYPADLTDSLATPA	58
BAM67139.1          --MTK---QFG-RNLAKRLRMAALSFSLLASSVSMIGAAQ---------AASAGAAPADG	45
WP_088042647.1      ---------MK-R--KKKSSWLSIFFTTALLLSTFFGNMSG--------PLVAKADSNPN	40
WP_068776920.1      ---------MR-S-KRVKAALVGLLAVL-CTFTPLLGA-GG--------SPVQAAAADSS	39
CHI                 --------------MKYLLPTAAAGLLL-LAAQPAMAM-DI-----------GINSDPEP	33
WP_103996460.1      ---------MK-SNKKIKMTWLSFFALL-LALLPLFNT-GA-----------GRVSAAEP	37
WP_009676326.1      ---------MK-SNKKIKMTWLGFFALL-LALLPLFNT-GA-----------GRASAAEP	37
WP_042235807.1      ---------MK-SNKRIKMTWLGFFALL-LALLPLFNT-GA-----------GTASAAEP	37
BAC76694.1          ---------MK-SNKRIKMTWLGFFALL-LALLPLFNT-GA-----------GTASAAEP	37
                                          .

WP_138185770.1      YKSVGYYTSWGIYGRNYQVTDIDASKLTHLNYSFFDICWNGIHGNPSTSSDNPNKKTWSC	118
BAM67139.1          YKVVGYFTSWGIYGRNFQVSDIDASKLTHINYAFADICWGGRHGNPSP--DSPNKTTWSC	103
WP_088042647.1      YKVIGYFPSWGIYGRNFQVENIDGSKLTHINYAFADICWNGIHGNPSP--DSPNKATWNC	98
WP_068776920.1      QKIVGYFTSWGIYGRNYKVPDIDATKLTHLNYAFADICWGGRHGNPSP--DSPNKNTWSC	97
CHI                 AKIVGYFTSWGIYGRNYQVKDIDGSKMTHLNYAFADICWGGVHGNNST--DSPNKQTWSC	91
WP_103996460.1      SKIVGYFTSWGIYGRNYQVKDIDGSKITHLNYAFADICWGGVHGNNST--DSPNKQTWSC	95
WP_009676326.1      AKIVGYFTSWGIYGRNYQVKDIDGSKLTHLNYAFADICWGGVHGNNST--DSPNKQTWSC	95
WP_042235807.1      AKIVGYFTSWGIYGRNYQVKDIDGSKMTHLNYAFADICWGGVHGNNST--DSPNKQTWSC	95
BAC76694.1          GKIVGYFTSWGIYGRNYQVKDIDGSKMTHLNYAFADICWGGVHGNNST--DSPNKQTWSC	95
                     * :**: ********::* :**.:*:**:**:* ****.* *** *   *.*** **.*

WP_138185770.1      TDPGVPLQKGSVPTGAIVLGEPWADVNTNFSGALNIPYEDCQK-GKCGNIARLKQLKSKN	177
BAM67139.1          TDAAVPLQRGSVPDGTIVLGEPWADVNQTVPGK---TYSECQE-GGCGNFERFRQLKAAN	159
WP_088042647.1      KDSTVPLQNKDVPNGTIVLGEPWADVSKSYPGK---IWSDCEQ-AKCGNIGKLNDLKVQY	154
WP_068776920.1      TDPAVPLQNKQVPNGTIVVGEPWADVNTPYGSY---TYDECNTKALCGNFAAMRDLKKKN	154
CHI                 TDSHVPLQSKSVPNGTIVLGEPWADVNTPYSGY---SYEECDQKALCGNFAGLRDLKKKN	148
WP_103996460.1      TDSHVPLQSKSVPNGTIVLGEPWADVNTPYAGY---SYEECDQKALCGNFAGLRDLKKKN	152
WP_009676326.1      TDSHVPLQSKSVPNGTIVLGEPWADVNTPYAGY---TYEECDQKALCGNFAGLRDLKKKN	152
WP_042235807.1      TDSHVPLQSKSVPNGTIVLGEPWADVNTPYSGY---SYEECDQKALCGNFAGLRDLKKKN	152
BAC76694.1          TDSHVPLQSKSVPNGSIVLGDAWADVNTPYSGY---SYEECDQKALCGNFAGLRDLKKKN	152
                    .*  ****  .** *:**:*: ****.    .     :.:*:  . ***:  :.:**

WP_138185770.1      PHLKTLFSVGGWTWSNRFSDVAANEQYRVNFAKSAVDAIREYGFDGIDIDWEYPGVEGIK	237
BAM67139.1          PHLKTIISVGGWTWSNRFSDVAASAATRQTFAKSAVQFLRTYGFDGVDLDWEYPVSGGLT	219
WP_088042647.1      PHLKTIISIGGWTWSNRFSDVAADPATRLVFAKSAVEFIRTYGFDGVDLDWEYPVSGGLS	214
WP_068776920.1      PSLKTLISVGGWTWSNRFSETAASAATRETFANSAVEFIRAYGFDGVDLDWEYPVAGGLA	214
CHI                 PSLKTLISVGGWTWSNRFSDVAANAATRETFANSAVEFIRTYGFDGVDLDWEYPVAGGLS	208
WP_103996460.1      PSLKTLISVGGWTWSNRFSETAASAATRETFANSAVEFIRTYGFDGVDLDWEYPVAGGLS	212
WP_009676326.1      PSLKTLISVGGWTWSNRFSDVAANATTRETFANSAVEFIRTYGFDGVDLDWEYPVAGGLS	212
WP_042235807.1      PSLKTLISVGGWTWSNRFSDVAANAATRETFANSAVEFIRTYGFDGVDLDWEYPVAGGLS	212
BAC76694.1          PSLKTLISVGGWTWSNRFSDVAANAATRETFANSAVEFIRTYGFDGVDLDWEYPVAGGLS	212
                    * ***::*:**********:.**.   *  **:***: :* *****:*:*****   *:

WP_138185770.1      GNSYSPNDKYNFTKLLAEVRKQIDAAGAQDGKHYLLTIATGASQRYIDNAEMDKVMQLVD	297
BAM67139.1          GNSYRPADKQNYTLLLQDIRNELDAAGKQDGKRYLLTIASGASQNYANNTELSKISSILD	279
WP_088042647.1      GNSYHPSDKQNYTLLLQAIRTELNKAELEDGKDYLLTIASGVSANYVTNTELDKISQTVD	274
WP_068776920.1      GNTYSPSDKQNYTLLLQKVREKLDAAGAQDGKKYLLTIASGASQKYADNTQLAEIAKVLD	274
CHI                 GNTYSPADKQNYTLLLKKVREKLDAAGTADGKKYLLTIASGASQKFANNTELSEIAKTVD	268
WP_103996460.1      GNTYSPADKQNYTLLLKKVREKLDAAGTADGKKYLLTIASGASQKYANNTELSEIAKTVD	272
WP_009676326.1      GNTYSPADKQNYTLLLKKVREKLDAAGTADGKKYLLTIASGASQKYANNTELSEIAKTVD	272
WP_042235807.1      GNTYSPADKQNYTLLLKKVREKLDAAGTADGKKYLLTIASGASQKFANNTELSEIAKTVD	272
BAC76694.1          GNTYSPADKQNYTLLLKKVREKLDAAGTADGKKYLLTIASGASQKFANNTELSEIAKTVD	272
                    **:* * ** *:* **  :* ::: *   *** ******:*.* .:  *::: :: . :*

WP_138185770.1      FINIMTYDFHGGWEKATNHNSALYGDPRDPAVA--TNFYVDGAIQVYERAGVDLSKVVMG	355
BAM67139.1          WINIMTYDFHGSWEKQTGFNAPLYSDPRDPADA--TKFYVDGAVNIYKQNGVPADKIVLG	337
WP_088042647.1      WINLMSYDFHGGWDTKTNHNAALYPVPNDPDKN--LGFTIDEAVTRYAQAGVPMNKLVMG	332
WP_068776920.1      WINIMTYDFHGGWETETNHNAALYVDPNDPTVGDKRKYNTNDAVQIYLNEGVPANKIVLG	334
CHI                 WINIMTYDFHGGWEKSTNHNAALYPDPNDPSTGDIKKYNTSDAIDIYFQSGVPANKLVLG	328
WP_103996460.1      WINIMTYDFHGGWEKSTNHNAALYPDPNDPSTGEIKKFNTSDAIDIYFQSGVPANKLVLG	332
WP_009676326.1      WINIMTYDFHGGWEKSTNHNAALYPDPNDPSTGEIKKFNTSDAIDIYFQSGVPANKLVLG	332
WP_042235807.1      WINIMTYDFHGGWEKSTNHNAALYPDPNDPSTGDIKKYNTSDAIDIYFQSGVPANKLVLG	332
BAC76694.1          WINIMTYDFHGGWEKSTNHNAALYPDPNDPSTGDIKKYNTSDAIDIYFQSGVPANKLVLG	332
                    :**:*:*****.*:. *..*: **  *.**       :  . *:  * . **  .*:*:*
```

```
WP_138185770.1      LPIYGVGWKGCAAGPNNDGLYQACKGGWDGNVTPSGTWDTWDAGATGVFDYGDLAANYVN	415
BAM67139.1          LAFYGRGWKGCEAGAAGDGLYQACKGGWDGSTVPAGTWDDWASGPSGNFDYGDLAANYVN	397
WP_088042647.1      LPFYGRAWKGVAN--ANNGEYQSITPGFDGTTVPMGTWDDYSSGATGVFDYGDIAANYVN	390
WP_068776920.1      LAFYGKGWKGCAPGPNNDGQYQKCTPGWDGSTLPTGTWDDWTSGNSGTFDYGDLAANYVG	394
CHI                 LPFYGKGWKGCPPGPNNDGQYQTCVGGWDGNVLPTGTWDDWASGNSGTFDYGDIMANYVN	388
WP_103996460.1      LPFYGKGWKGCPPGPNNDGQYQTCVGGWDGNVLPTGTWDDWASGNSGTFDYGDIMANYVN	392
WP_009676326.1      LPFYGKGWKGCPPGPNNDGQYQTCVGGWDGSVLPTGTWDDWASGNSGTFDYGDIMANYVN	392
WP_042235807.1      LPFYGKGWKGCPPGPNNDGQYQTCVGGWDGNVLPTGTWDDWASGNSGTFDYGDIMANYVN	392
BAC76694.1          LPFYGKGWKGCPPGPNNDGQYQTCVGGWDGNVLPTGTWDDWASGNSGTFDYGDIMANYVN	392
                    * :** .***      .:* **    *:**.. * **** : :* :* *****: ****.

WP_138185770.1      KNGYQRYWNDTAKAPYLFNAATGQFMAYDDIQSIGYKTSYIKSKGLGGAMYWDLSSDCRT	475
BAM67139.1          KNGYTRYWNDYAKVPYVYNPTNGVFIGYDDVESIGHKTNYIKQQGLGGAMFWEASNDCRT	457
WP_088042647.1      KNGFTRYWNNTAKVPFLYNASTGVFMTYDDTESFGYKTDYIKSKGLAGGMFWELSSDCRT	450
WP_068776920.1      KNGYTRYWNDTTKTPYLFNPTNGVFISYDDIQSIGAKTAYIKSKGLGGAMFWETSSDCRT	454
CHI                 KNGFTRYWNDTTKTPYLFNPTSGTFISYEDTQSIAAKTAYIKIKGLAGAMFWETSSDCRT	448
WP_103996460.1      KNGFTRYWNDTTKTPYLFNPTSGTFISYEDTQSIAAKTAYIKSKGLAGAMFWETSSDCRT	452
WP_009676326.1      KNGFTRYWNDTTKTPYLFNPTSGTFISYEDTQSIAAKTAYIKSKGLAGAMFWETSSDCRT	452
WP_042235807.1      KNGFTRYWNDTTKTPYLFNPTSGTFISYEDTQSIAAKTAYIKSKGLAGAMFWETSSDCRT	452
BAC76694.1          KNGFTRYWNDTTKTPYLFNPTSGTFISYEDTQSIAAKTAYIKSKGLAGAMFWETSSDCRT	452
                    ***: ****: :*.*:::* :.* *: *:* :*:. ** *** :**.*.*:*: *.****

WP_138185770.1      SSKYTCTGQKMIDKVAADLGINA--NDPGNLPPTVPQGLSVVQVTYSEVAFKWNPSTSNV	533
BAM67139.1          SSKFACTGPKLLDKIATDLQGGK--VPSDTVPPTAPGNLT-AAKTTTTVTLNWEGSTDNY	514
WP_088042647.1      SSKYTCTGPKLIDKLASDLLGGGVSIPPDTQAPTVVSNLNSPSKTSSSIQLSWTASTDNV	510
WP_068776920.1      SPKFSCTT-KLLDKVASDLLTGGP-VQPDTQAPTAATNLTSPSKTANSVALSWTAATDNV	512
CHI                 SPKFSCTI-KLLDKVAADLMS--P-AVPDTQAPTAVTNLVSTGKTSTSVTLSWTASTDNV	504
WP_103996460.1      SPKFSCTV-KLLDKVAADLQT--P-ATPDTQAPTAVTNLISTGKTATSVALSWTAATDNV	508
WP_009676326.1      SPKFSCTT-KLLDKVAADLLS--P-AVPDTQAPTAVTNLVSTGKTSTSVTLSWTAATDNV	508
WP_042235807.1      SPKFSCTI-KLLDKVAADLMS--P-AVPDTQAPTAVTNLVSTGKTSTSVTLSWTASTDNV	508
BAC76694.1          SPKFSCTI-KLLDKVAADLMS--P-AVPDTQAPTAVTNLVSTGKTSTSVTLSWTASTDNV	508
                    * *::**  *::**:*:**         ..  **.  .*     * . : :.*  :*.*

WP_138185770.1      GVSGYDVYKDGSLVLSIPQTEATIKGLTENTDYRFTVKAKDTLGNVSEASAPLLVRTAKK	593
BAM67139.1          GVTGYEVYNGSTLVGITTAKTYTVSGLTPETAYTFKVLAKDAAGNKSAASQVTVTTDK--	572
WP_088042647.1      GVTSYEISYGSTKLTSTT-TSLNVTGLQADTNYTFSVTAKDAAGNTSQAASITVKTNA--	567
WP_068776920.1      GVAGYEVSYGTNKVTVTG-TAANITGLLPSTAYTFTVKAKDAAGNLSAPVSITVTTNAGG	571
CHI                 GVAGYEVSYGTTKVNVPG-TTANITGLTANTAYTFTVKAKDAAGNVSAPASVTVTTDGGT	563
WP_103996460.1      GVAGYEVSYGTTKVNATG-TTANITGLTAETAYTFTVKAKDAAGNVSAPASVTVTTASGT	567
WP_009676326.1      GVAGYEVSYGTTKVNVTG-TTANITGLTANTAYTFTVKAKDAAGNVSAPASVTVTTDGGT	567
WP_042235807.1      GVAGYEVSYGTTKVNVPG-TTANITGLTANTAYTFTVKAKDAAGNVSAPASVTVTTDGGT	567
BAC76694.1          RVAGYEVSYGTTKVNVPG-TTANITGLTANTAYTFTVKAKDAAGNVSAPASVTVTTDGGT	567
                     *:.*::  . . :     .  .:.**  .* * *.* ***: ** *      :

WP_138185770.1      PPVDIDPPSAPTQVVVTGKSNTTVSLSWAPSTSKAGVDRYEVALTGSTVAQAKGTTVTVT	653
BAM67139.1          VVPDNVAPSVPTNVQAASKTDTSVNLTWTASTDNIGVTGYDVYKDGVLAGTSATTSYAVT	632
WP_088042647.1      QSTDTQAPTAVTNLSSPSKTTNSVQLSWTSATDNVGVTGYEISYGSTKL-STTATSINVT	626
WP_068776920.1      AQPDTQAPTAVTNLVSTGKTANSVALSWTAATDNVGVTGYEVAYGTNVV-NAAGTSTNVT	630
CHI                 TTPDTQAPTAVTNLVSTGKTSTSVALSWTAATDNIGVTGYDVTYGTKTV-STTATSLNVT	622
WP_103996460.1      TTPDTQAPTAVTNLVSTGKTSTSAALSWTAATDNVGVTGYDVTYGTKTV-STTATSLNVT	626
WP_009676326.1      TTPDTQAPTAVTNLVSTGKTSTSAALSWTAATDNVGVTGYDVTYGTKTV-STTATSLNVT	626
WP_042235807.1      TTPDTQAPTAVTNLVSTGKTSTSVALSWTAATDNIGVTGYDVTYGTKTV-STTATSLNVT	626
BAC76694.1          TTPDTQAPTAVTNLVSTGKTSTSVALSWTAATDNIGVTGYDVTYGTKTV-STTATSLNVT	626
                       *   *:. *::   .*: .:. *:*: :*.: **  *::         :  *:  **

WP_138185770.1      SLSPETSYTFRITAVDTVGNRSEPSEPITVVTDKTAK---SDVS-FIFAITSDWGTGYNF	709
BAM67139.1          GLTANTSYSFTVKAKDAAGNASAASTAVIVTTNAGGVVKATGVPAAPAVTHDNWDNDGNY	692
WP_088042647.1      GLQANTNYTFAVTAKDAAGNTSQPTSITV-QTNTQ-GGTTSQVNVT-FTISSDWGTGFNY	683
WP_068776920.1      GLQPNTAYTFTVKAKDAAGNLSAPASVTV-TTDTGTTNPGPGVETT-FKVTSDWGTGYNY	688
CHI                 DLTPSTAYTFTVKAKDAAGNVSAPASVTV-TTDA-ATNPGSPVQPT-FTVTSDWGTGYNF	679
WP_103996460.1      DLTPNTAYTFTVKAKDAAGNVSAPAAVSV-TTDP-ATNPGSPVQPT-FTVTSDWGTGYNF	683
WP_009676326.1      DLTPNTAYTFTVKAKDAAGNISAPASVTV-TTDP-ATNPGSPVQPT-FTVTSDWGTGYNF	683
WP_042235807.1      DLTPSTAYTFTVKAKDAAGNVSAPASVTV-TTDA-ATNPGSPVQPT-FTVTSDWGTGYNF	683
BAC76694.1          DLTPSTAYTFTVKAKDAAGNVSAPASVTV-TTDA-ATNPGSPVQPT-FTVTSDWGTGYNF	683
                    .*  .* *:* :.* *:.** *  :      *:         *        .:*... *:

WP_138185770.1      QGTLTNNGSADITSWRLEFDYSG-SISQIWDARIVSRNGNH------YIIESAGWNNSIP	762
BAM67139.1          NITFNIWWGNNGSSWKLYENN-----QVVFTESLVDNSPNAQTAKKEFTGKAKGTY-KYK	746
WP_088042647.1      DLKIKNNGTAPINNWRLEFDYTG-NITTIWDAKIVSKTGNH------YVIESAGWNSVIA	736
```

```
WP_068776920.1      SFTIKNTGTTPITNWKLEFDYAGGDINAIWDATIVSKTNNH------YVIKGSGWNSTLQ        742
CHI                 SFSIKNTGTTPITNWKLEFDYTG-SITSVWDASIVSSANNH------FVIKGAGWNNTLQ        732
WP_103996460.1      SFSIKNTGTTPITNWKLEFDYTG-SITSVWDASIVSSANNH------FVIKGAGWNNTLQ        736
WP_009676326.1      SFSIKNTGTTPITNWKLEFDYTG-SITSVWDASIVSSANNH------FVIKGAGWNNTLQ        736
WP_042235807.1      SFSIKNTGTTPITNWKLEFDYTG-SITSVWDASIVSSANNH------FVIKGAGWNNTLQ        736
BAC76694.1          SFSIKNTGTTPITNWKLEFDYTG-SITSVWDASIVSSANNH------FVIKGAGWNNTLQ        736
                    . .:.       ..*:*  :        ::   :*.   *       :  :. *

WP_138185770.1      SKGSVSFGGGGSPGGNSSQPTNIVVTWK--------        790
BAM67139.1          VELTNSFGTSTS--------QEVTVTVN--------        766
WP_088042647.1      AGGTVSFGGGGSGASS-PQIQNAVVTGN--------        763
WP_068776920.1      PGATVTFGGGGLVKST-P--TNITVTSN--------        767
CHI                 PGATVTFGGAGLVKAQ-P--TNIVVTGSLEHHHHHH        765
WP_103996460.1      PGATVTFGGAGLVKAQ-P--TNIVVTGS--------        761
WP_009676326.1      PGATVTFGGAGLVKAQ-P--TNIVVTGS--------        761
WP_042235807.1      PGATVTFGGAGLVKAQ-P--TNIVVTGS--------        761
BAC76694.1          PGATVTFGGAGLVKAQ-P--TNIVVTGS--------        761
                       : :** .           : .** .
```
