# Supplementary material for: Cloning, expression and characterization of a chitinase from Paenibacillus chitinolyticus strain UMBR 0002
Source: PeerJ. 2020 May 5;8:e8964. doi: 10.7717/peerj.8964 (PMC7207210; doi:10.7717/peerj.8964)
Supplement: Supplemental Information 10 [file peerj-08-8964-s010.pdf]

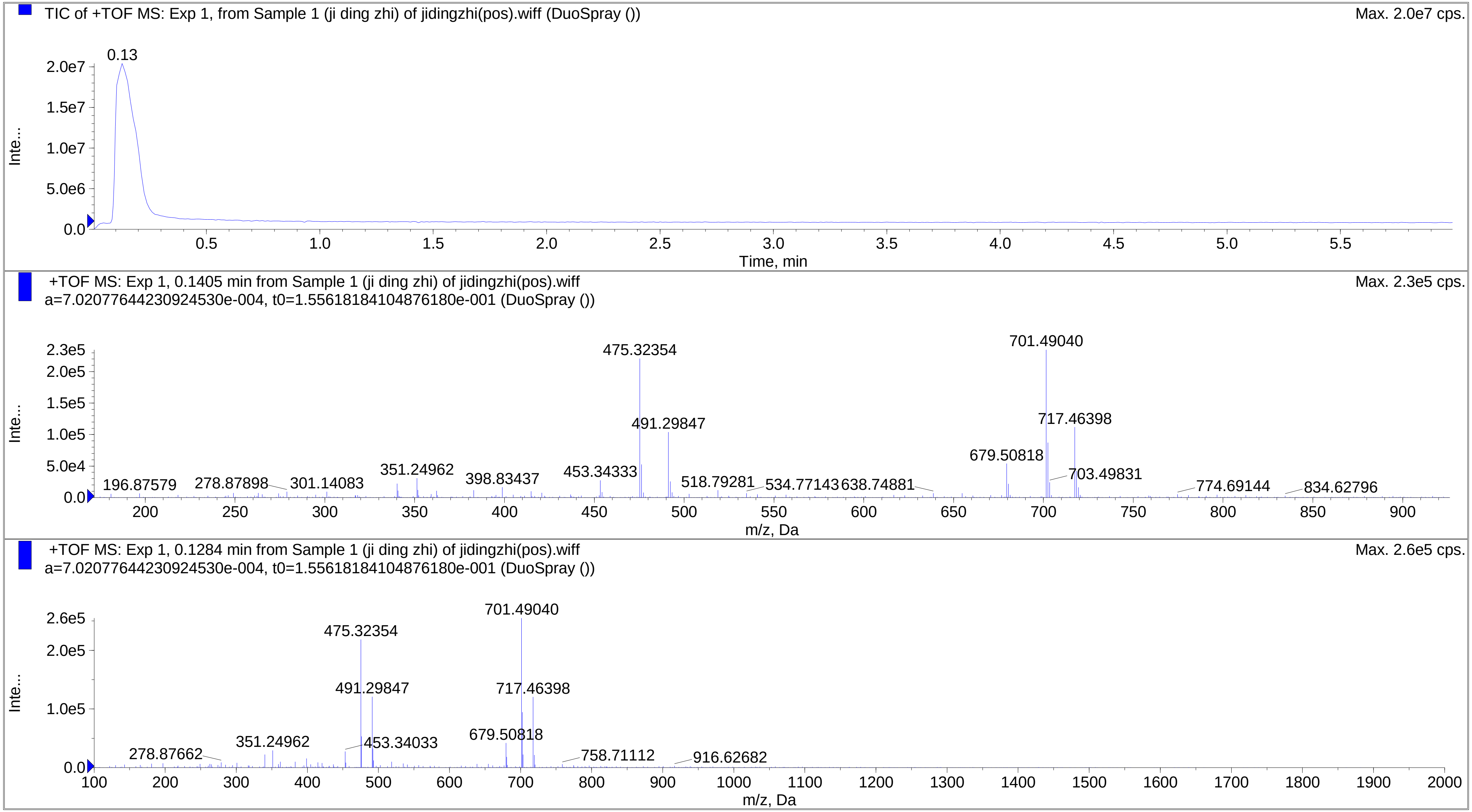
TIC of +TOF MS: Exp 1, from Sample 1 (ji ding zhi) of jidingzhi(pos).wiff (DuoSpray ())
Max. 2.0e7 cps.
0.13
Inte...
Time, min
+TOF MS: Exp 1, 0.1405 min from Sample 1 (ji ding zhi) of jidingzhi(pos).wiff
a=7.02077644230924530e-004, t0=1.55618184104876180e-001 (DuoSpray ())
Max. 2.3e5 cps.
475.32354
701.49040
491.29847
717.46398
679.50818
703.49831
196.87579
278.87898
301.14083
351.24962
398.83437
453.34333
518.79281
534.77143
638.74881
774.69144
834.62796
m/z, Da
+TOF MS: Exp 1, 0.1284 min from Sample 1 (ji ding zhi) of jidingzhi(pos).wiff
a=7.02077644230924530e-004, t0=1.55618184104876180e-001 (DuoSpray ())
Max. 2.6e5 cps.
701.49040
475.32354
491.29847
717.46398
351.24962
453.34033
679.50818
278.87662
758.71112
916.62682
m/z, Da
