# Supplementary material for: Cloning, expression and characterization of a chitinase from Paenibacillus chitinolyticus strain UMBR 0002
Source: PeerJ. 2020 May 5;8:e8964. doi: 10.7717/peerj.8964 (PMC7207210; doi:10.7717/peerj.8964)
Supplement: Supplemental Information 11 [file peerj-08-8964-s011.pdf]

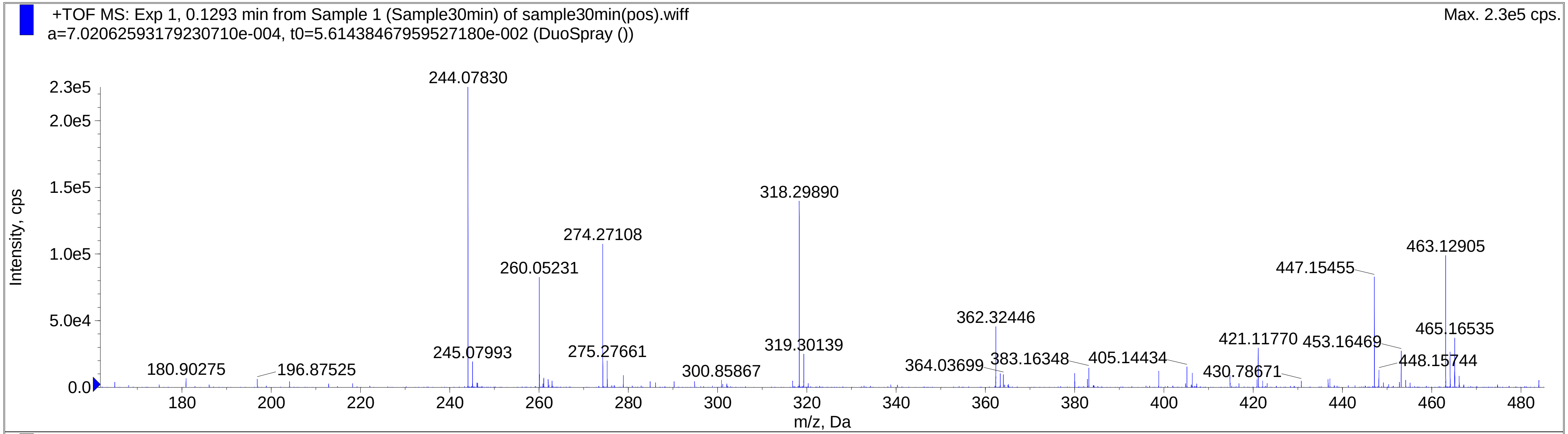
+TOF MS: Exp 1, 0.1293 min from Sample 1 (Sample30min) of sample30min(pos).wiff
a=7.02062593179230710e-004, t0=5.61438467959527180e-002 (DuoSpray ())
Max. 2.3e5 cps.
Intensity, cps
m/z, Da
244.07830
318.29890
274.27108
260.05231
463.12905
447.15455
362.32446
465.16535
421.11770
453.16469
319.30139
245.07993
275.27661
180.90275
196.87525
300.85867
364.03699
383.16348
405.14434
430.78671
448.15744

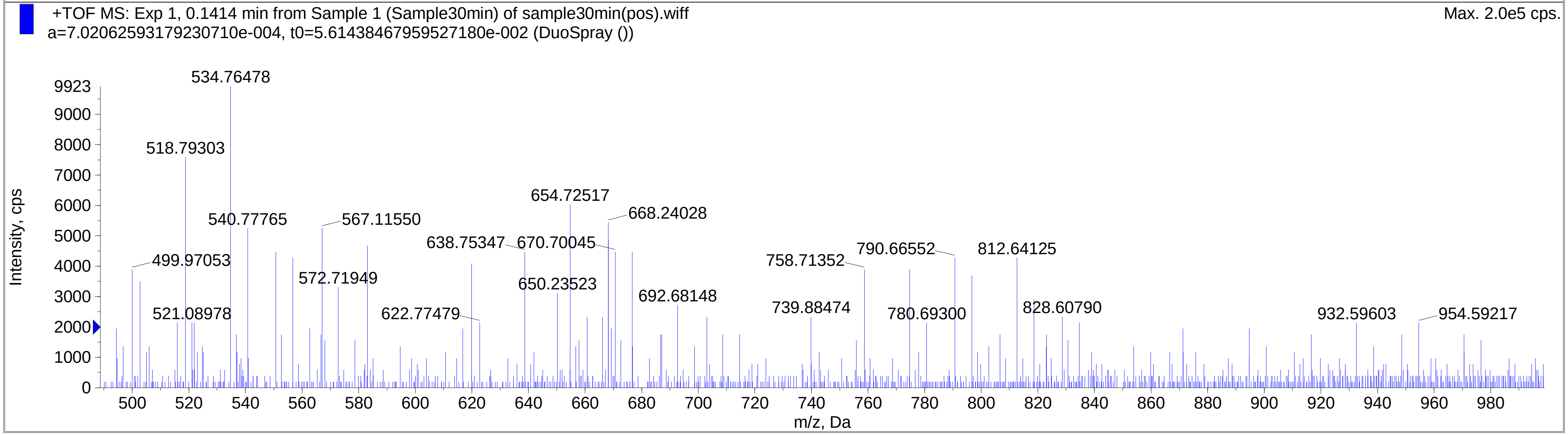
+TOF MS: Exp 1, 0.1414 min from Sample 1 (Sample30min) of sample30min(pos).wiff
a=7.02062593179230710e-004, t0=5.61438467959527180e-002 (DuoSpray ())
Max. 2.0e5 cps.
Intensity, cps
m/z, Da
534.76478
518.79303
654.72517
540.77765
567.11550
668.24028
638.75347
670.70045
499.97053
790.66552
812.64125
758.71352
572.71949
650.23523
692.68148
521.08978
622.77479
739.88474
780.69300
828.60790
932.59603
954.59217
