# Supplementary material for: Cloning, expression and characterization of a chitinase from Paenibacillus chitinolyticus strain UMBR 0002
Source: PeerJ. 2020 May 5;8:e8964. doi: 10.7717/peerj.8964 (PMC7207210; doi:10.7717/peerj.8964)
Supplement: Supplemental Information 12 [file peerj-08-8964-s012.pdf]

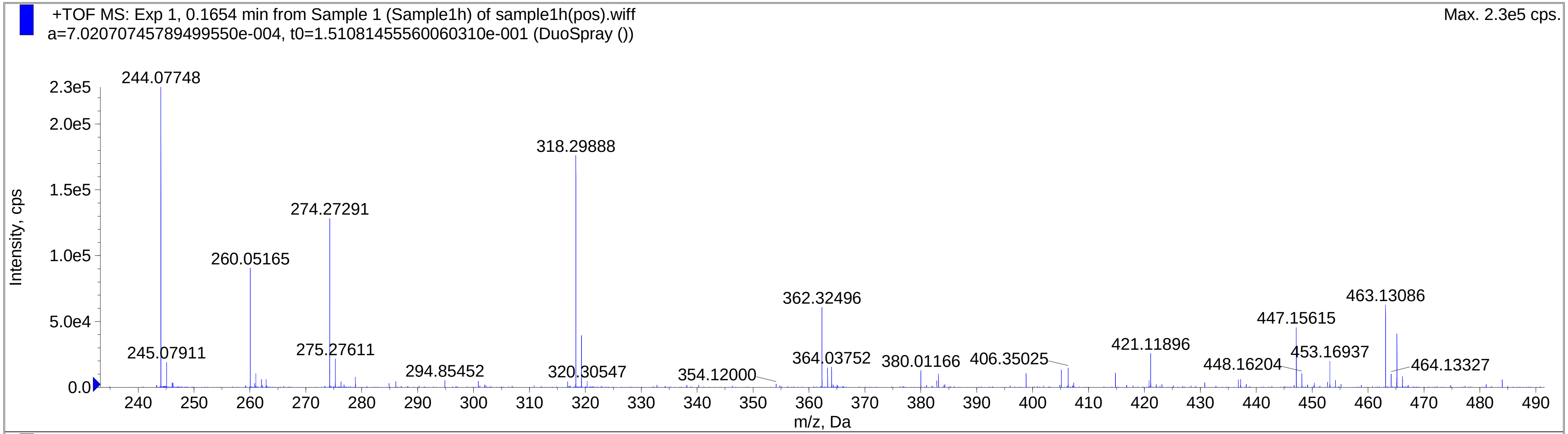
+TOF MS: Exp 1, 0.1654 min from Sample 1 (Sample1h) of sample1h(pos).wiff
a=7.02070745789499550e-004, t0=1.51081455560060310e-001 (DuoSpray ())
Max. 2.3e5 cps.
Intensity, cps
m/z, Da
244.07748
245.07911
260.05165
274.27291
275.27611
294.85452
318.29888
320.30547
354.12000
362.32496
364.03752
380.01166
406.35025
421.11896
447.15615
448.16204
453.16937
463.13086
464.13327

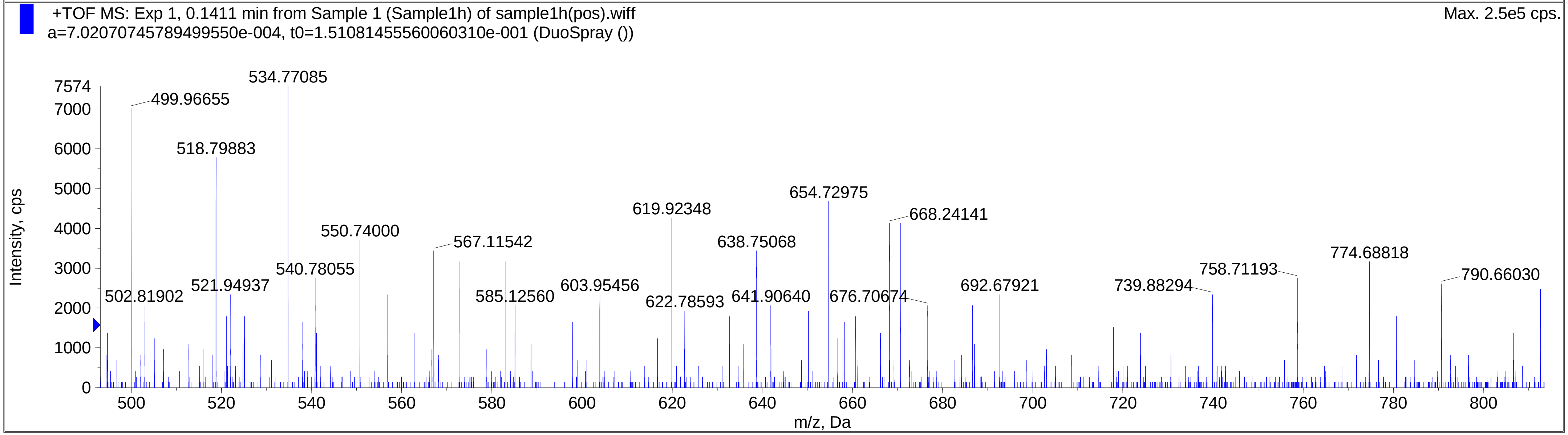
+TOF MS: Exp 1, 0.1411 min from Sample 1 (Sample1h) of sample1h(pos).wiff
a=7.02070745789499550e-004, t0=1.51081455560060310e-001 (DuoSpray ())
Max. 2.5e5 cps.
Intensity, cps
m/z, Da
499.96655
502.81902
518.79883
521.94937
534.77085
540.78055
550.74000
567.11542
585.12560
603.95456
619.92348
622.78593
638.75068
641.90640
654.72975
668.24141
676.70674
692.67921
739.88294
758.71193
774.68818
790.66030
