# Supplementary material for: Cloning, expression and characterization of a chitinase from Paenibacillus chitinolyticus strain UMBR 0002
Source: PeerJ. 2020 May 5;8:e8964. doi: 10.7717/peerj.8964 (PMC7207210; doi:10.7717/peerj.8964)
Supplement: Supplemental Information 13 [file peerj-08-8964-s013.pdf]

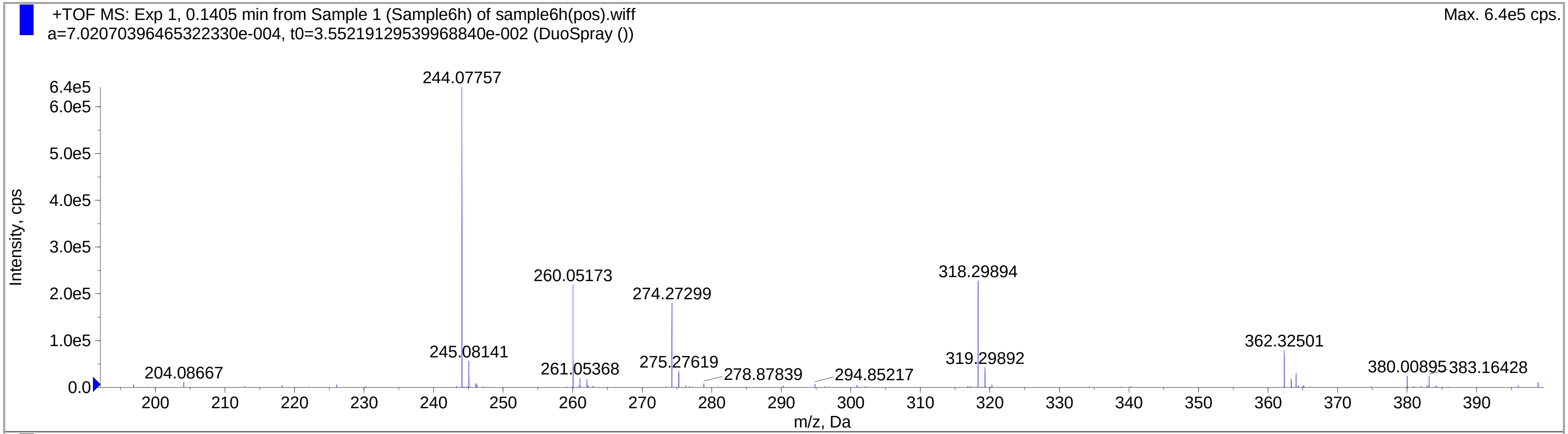
+TOF MS: Exp 1, 0.1405 min from Sample 1 (Sample6h) of sample6h(pos).wiff
a=7.02070396465322330e-004, t0=3.55219129539968840e-002 (DuoSpray ())
Max. 6.4e5 cps.
Intensity, cps
m/z, Da
244.07757
260.05173
274.27299
318.29894
362.32501
245.08141
261.05368
275.27619
278.87839
294.85217
319.29892
204.08667
380.00895
383.16428

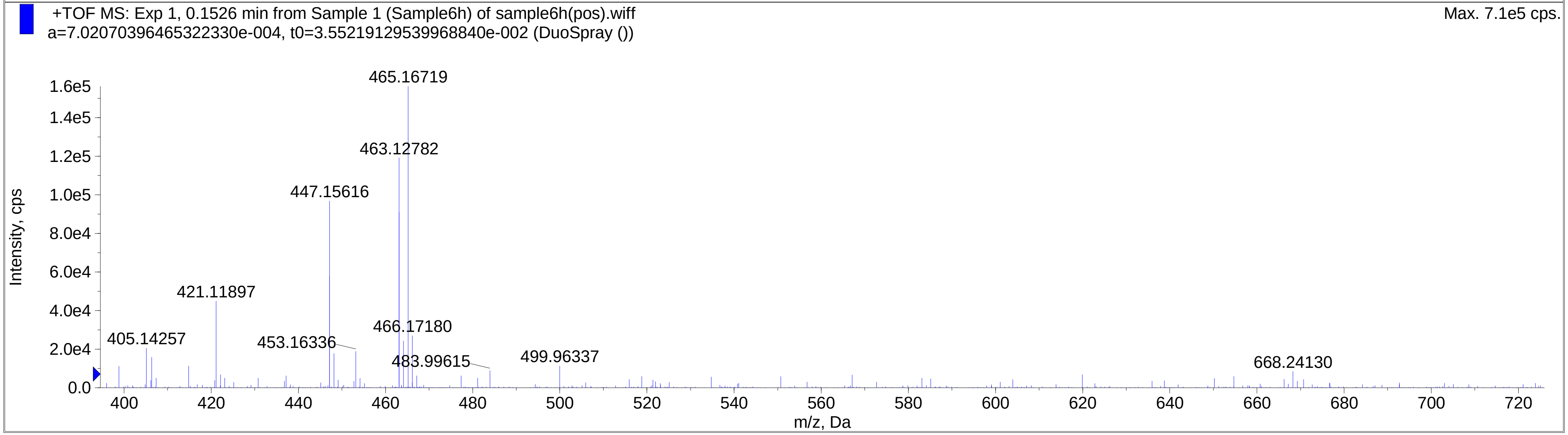
+TOF MS: Exp 1, 0.1526 min from Sample 1 (Sample6h) of sample6h(pos).wiff
a=7.02070396465322330e-004, t0=3.55219129539968840e-002 (DuoSpray ())
Max. 7.1e5 cps.
Intensity, cps
m/z, Da
465.16719
463.12782
447.15616
421.11897
405.14257
453.16336
466.17180
483.99615
499.96337
668.24130
