# Supplementary material for: Cloning, expression and characterization of a chitinase from Paenibacillus chitinolyticus strain UMBR 0002
Source: PeerJ. 2020 May 5;8:e8964. doi: 10.7717/peerj.8964 (PMC7207210; doi:10.7717/peerj.8964)
Supplement: Supplemental Information 14 [file peerj-08-8964-s014.pdf]

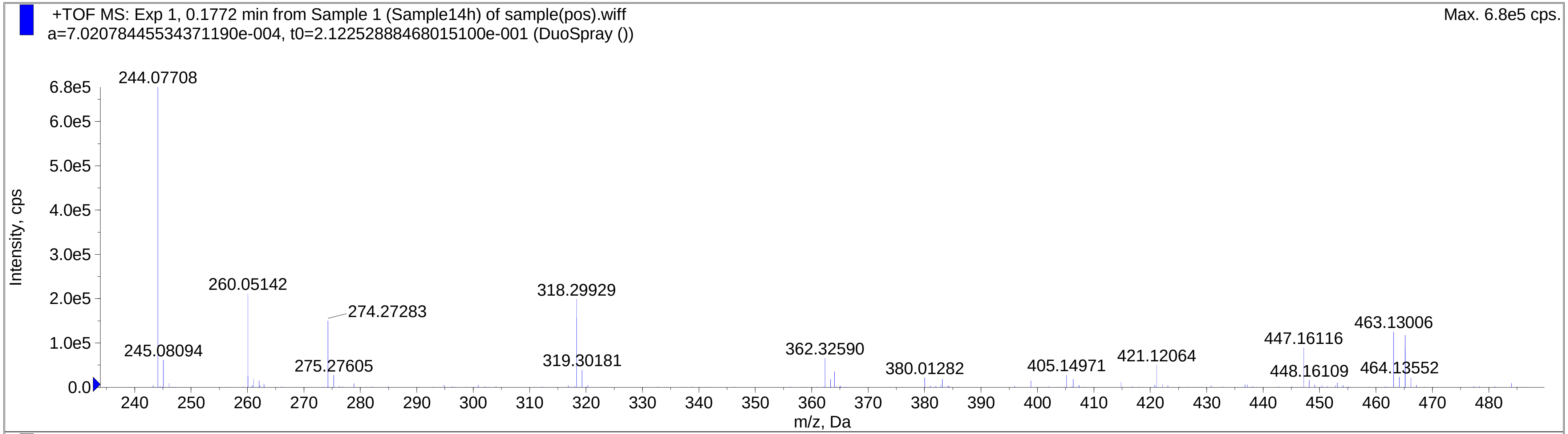
+TOF MS: Exp 1, 0.1772 min from Sample 1 (Sample14h) of sample(pos).wiff
a=7.02078445534371190e-004, t0=2.12252888468015100e-001 (DuoSpray ())
Max. 6.8e5 cps.
Intensity, cps
m/z, Da
244.07708
245.08094
260.05142
274.27283
275.27605
318.29929
319.30181
362.32590
380.01282
405.14971
421.12064
447.16116
448.16109
463.13006
464.13552

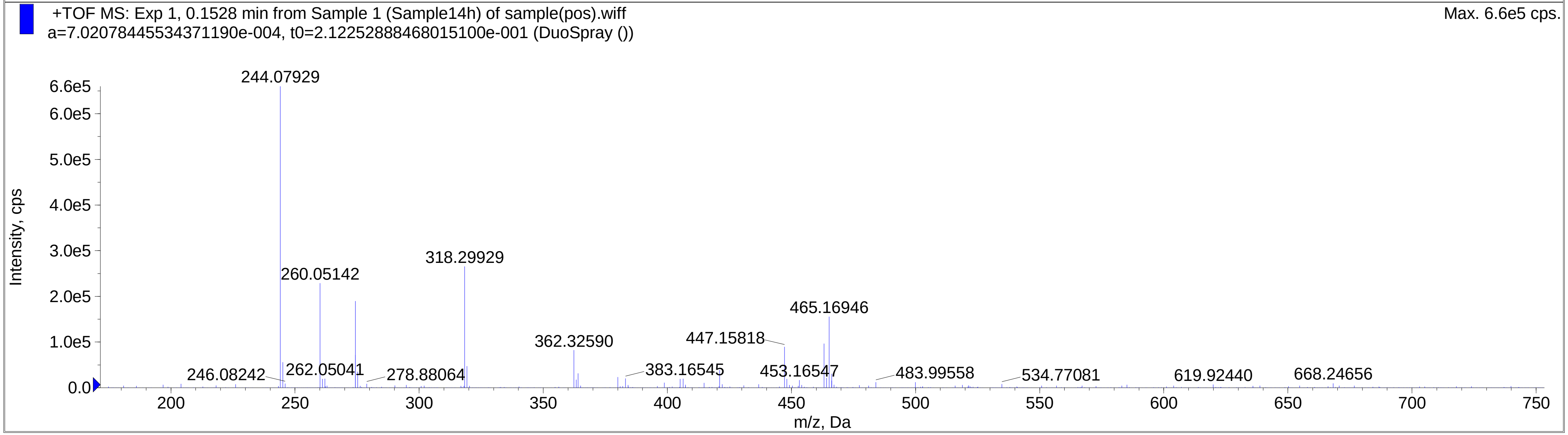
+TOF MS: Exp 1, 0.1528 min from Sample 1 (Sample14h) of sample(pos).wiff
a=7.02078445534371190e-004, t0=2.12252888468015100e-001 (DuoSpray ())
Max. 6.6e5 cps.
Intensity, cps
m/z, Da
244.07929
246.08242
260.05142
262.05041
278.88064
318.29929
362.32590
383.16545
447.15818
453.16547
465.16946
483.99558
534.77081
619.92440
668.24656
